# Supplementary material for: Phthalate Esters Metabolic Strain Gordonia sp. GZ-YC7, a Potential Soil Degrader for High Concentration Di-(2-ethylhexyl) Phthalate
Source: Microorganisms. 2022 Mar 17;10(3):641. doi: 10.3390/microorganisms10030641 (PMC8955600; doi:10.3390/microorganisms10030641)
Supplement: Supplementary file 1 [file microorganisms-10-00641-s001.zip › Supplementary Figures.pdf]

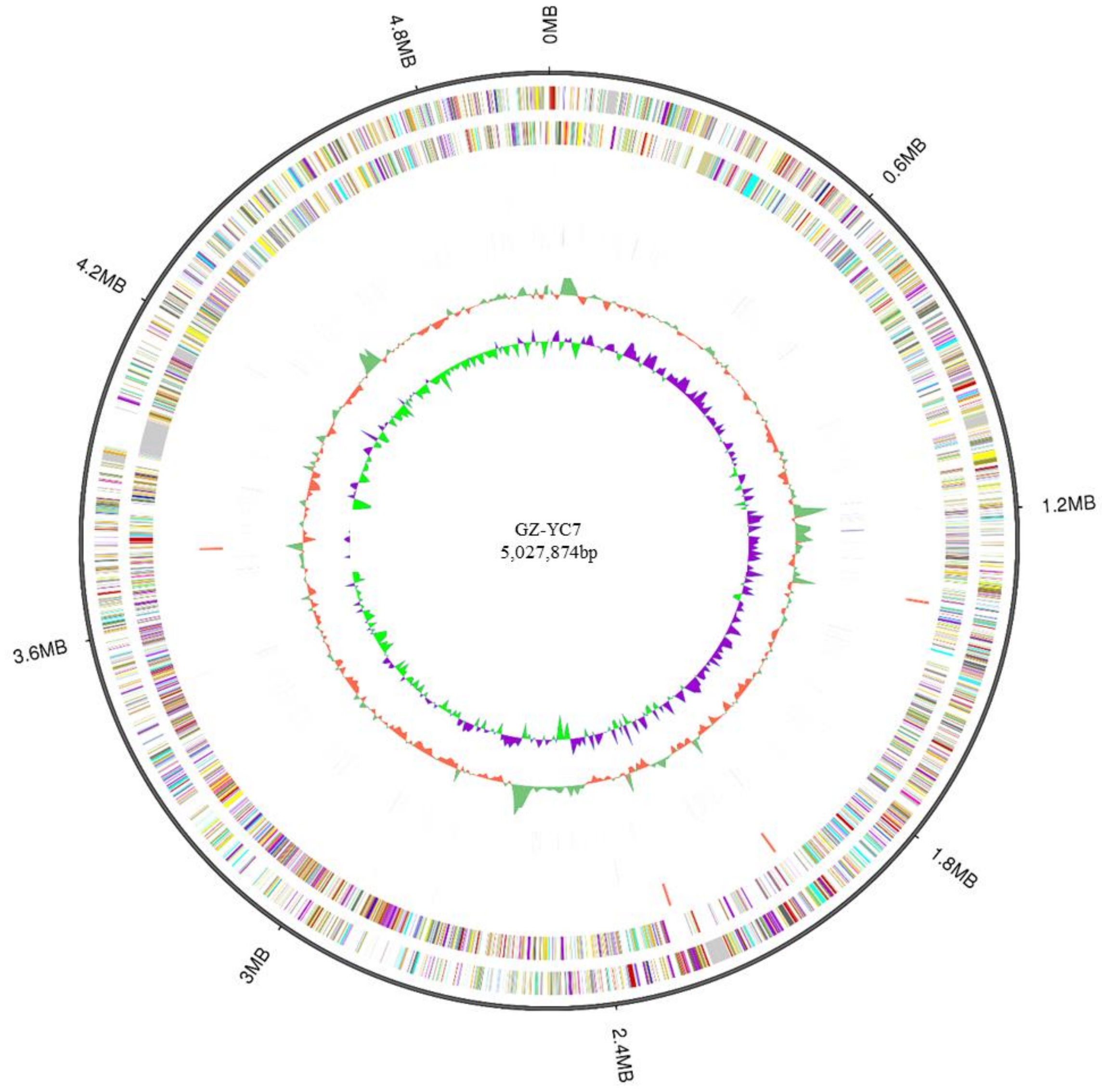

Figure S1 Circular genome map of *Gordonia* sp. GZ-YC7. The eight circles for chromosome from the outside to the center represent respectively genome size (circle1), forward strand gene (circle2), reverse strand gene (circle3), forward strand ncRNA (circle4), reverse strand ncRNA (circle5), repeat (circle6), GC content (circle7), GC-SKEW (circle8).

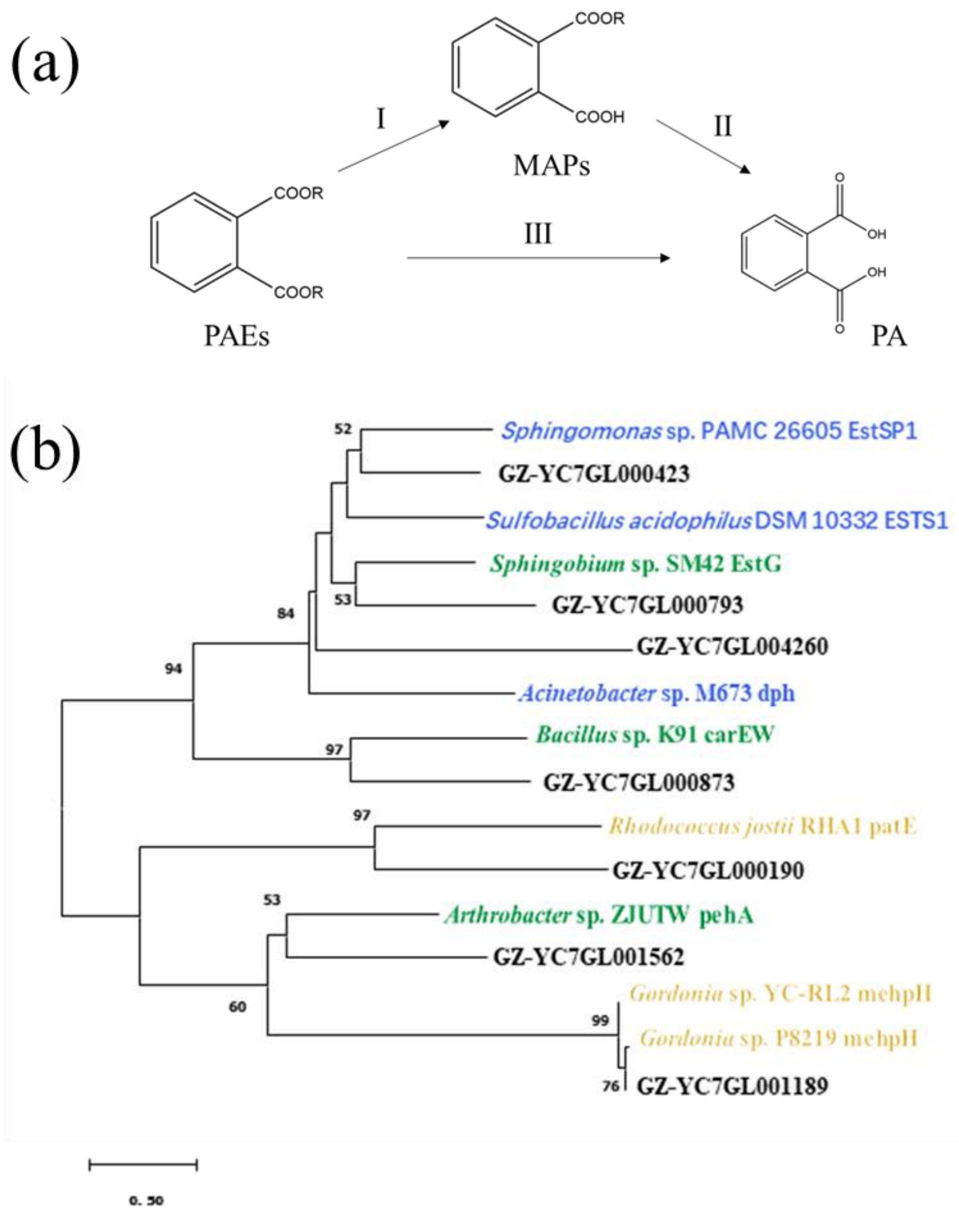

Figure S2. Catalytic process of PAEs by three types of esters (a); Phylogenetic tree analysis of seven putative esterase genes in GZ-YC7 and other reported esterase genes (b). The blue represents Type I esterase genes, the yellow represents Type II esterase genes, and the green represents Type III esterase genes. .

MPAs (Phthalate Monoesters); PA (Phthalic acid).
