# Supplementary material for: Phthalate Esters Metabolic Strain Gordonia sp. GZ-YC7, a Potential Soil Degrader for High Concentration Di-(2-ethylhexyl) Phthalate
Source: Microorganisms. 2022 Mar 17;10(3):641. doi: 10.3390/microorganisms10030641 (PMC8955600; doi:10.3390/microorganisms10030641)
Supplement: Supplementary file 1 [file microorganisms-10-00641-s001.zip › Supplementary Tables.pdf]

Table S1 Kinetic parameters of DEHP degradation

DEHP (mg/L)	Kinetic equations	$t_{1/2}$(d)	R^2
2000	$\ln C = -0.01934t + 7.6001$	1.49334	0.9107
1000	$\ln C = -0.03281t + 6.9013$	0.88025	0.9274
500	$\ln C = -0.06471t + 6.1809$	0.44632	0.9076
200	$\ln C = -0.06411t + 5.3237$	0.45049	0.8906

C: The concentration of DEHP at time t; $t_{1/2}$: half-life

Table S2 The whole genome sequencing and assembly related information of *Gordonia* sp. GZ-YC7

Platform	Insert Size (bp)	Reads Length (bp)	Raw Data (Mb)	Adapter (%)	Duplication (%)	Total Reads	Filtered Reads (%)	Low Quality Filtered Reads (%)	Clean Data (Mb)
DNBSEQ	350	(150:150)	1,314	0	0.02	8,764,210	0.07	0	1,308

Platform	Valid Zero-Mode Waveguides Number	Subreads Number	Subreads Total Bases (bp)	Subreads Mean Length (bp)	Subreads N50 (bp)	Subreads N90 (bp)	Subreads Max Length (bp)	Subreads Min Length (bp)
PacBio	34,772	248,485	2,525,005,289	10,161	11,060	6,917	150,259	2,000

Table S3 Gene numbers of each category

Category	Gene number
tRNA	51 (1.10 %)
5s rRNA	4 (0.09 %)
16s rRNA	4 (0.09 %)
23s rRNA	4 (0.09 %)
sRNA	9 (0.19 %)
VFDB	223 (4.79 %)
ARDB	7 (0.15 %)
TREMBL	4,476 (96.17 %)
CAZY	126 (2.70%)
IPR	3,870 (83.15 %)
Swiss-Prot	1,758 (37.77 %)
COG	3,463 (74.4 %)
CARD	1 (0.02 %)
GO	2,670 (57.37 %)
KEGG	2,400 (51.56 %)
NR	4.481 (96.28 %)
T3SS	1,026 (22.04 %)

Table S4 Esterase genes in previous publications

Type	Ester	Strains	Reference
I	*dph*	*Acinetobacter* sp. M673	[50]
I	*EstS1*	*Sulfobacillus acidophilus* DSM10332	[2]
I	*EstSP1*	*Sphingomonas glacialis* PAMC 26605	[45]
II	*mehpH*	*Gordonia alkanivorans* YC-RL2	[31]
II	*mehpH*	*Gordonia* sp. P8219	[44]
II	*patE*	*Rhodococcus jostii* RHA1	[46]
III	*EstG*	*Sphingobium* sp. SM42	[47]
III	*CarEW*	*Bacillus* sp. K91	[48]
III	*pehA*	*Arthrobacter* sp. ZJUTW	[30]
